# Reply: Matters Arising ‘Investigating sources of inaccuracy in wearable optical heart rate sensors’

**DOI:** 10.1038/s41746-021-00409-4

**Published:** 2021-02-26

**Authors:** Brinnae Bent, Oana M. Enache, Benjamin Goldstein, Warren Kibbe, Jessilyn P. Dunn

**Affiliations:** 1 Department of Biomedical Engineering, Duke University, Durham, NC USA; 2 Department of Biostatistics and Bioinformatics, Duke University School of Medicine, Durham, NC USA

**Keywords:** Peer review, Imaging and sensing

**Replying to**: Colvonen et al. *npj Digital Medicine* 10.1038/s41746-021-00408-5 (2021)

This is a response to the Matters Arising (MA) that examines our original article, ‘Investigating inaccuracies in wearable optical heart rate sensors’^1^. We performed this original study to address the concern that there was inadequate published research on the potential effect of skin tone on wearable device accuracy. The central hypothesis tested in the original study was that darker skin tones have decreased photoplethysmography-based heart rate measurement accuracy as compared with lighter skin tones. The MA suggests improvements surrounding two aspects of the original study: the sample size and the use of the Fitzpatrick skin tone (FP) scale to categorize skin tones. The original study was designed and powered according to the above hypothesis. We acknowledge that visual skin tone scales are imperfect, and that a study can never prove the null hypothesis to be true. We, too, encourage more work examining wearable device accuracy across skin tones. In this reply, we aim to address questions surrounding the sample size, covariates, and choice of skin tone scale in the original article.

The two overarching analyses planned for the original study were an Analysis of Variance (ANOVA) to test for a difference in means of heart rate accuracy between FP skin tone groups, and a mixed effects regression model to explore potential effects of wearable device type and activity type during wear. To achieve 80% power to reject the null hypothesis that there is no difference in heart rate accuracy between the six FP groups (∝ = 0.5), we concluded that 48 participants were needed overall, with eight participants in each of the skin tone categories for the ANOVA and 46 needed for the mixed effects model (f2 = 0.15). We acknowledge that power analyses are imperfect sample size calculation tools and that domain-knowledge based decisions must be made to define the parameters of the power analysis. Here, a medium effect size of 0.3 was chosen based on a pilot study examining differences in green light absorption across skin tones on the FP scale^2^.

In this study, we recruited and enrolled an approximately equal distribution of skin tones to meet our power requirements (with 7, 8, 10, 9, 9, and 10 participants, respectively, for FP groups 1–6). In the MA it is suggested that there should be an increased number of participants in FP6. Addressing concerns raised in the MA of greater heterogeneity in darker skin tone groups, we did not find differences in variance of heart rate measurements or their errors across skin tone groups (Fig. 2). Overall, we recommend a statistically-based justification for all sample size choices.

Given established effects of movement on PPG heart rate accuracy^3–6^, in the original study we comprehensively explored potential interaction between skin tone and level of activity (Fig 2). No interaction effects were found that could not be directly attributed to the differences among activity (Fig. 3). We also did not find any relationship between weight, BMI, and body fat percentage and heart rate accuracy or any interaction with skin tone.

Potential covariates proposed in the MA that were not measured in the original study include arm hair, sweat, and thickness of skin epidermis. We are aware that skin epidermal thickness can be measured with appropriate optical equipment^7^ and should be explored in future studies; however, the other factors are more difficult to quantify. Further, increasing the number of covariates measured increases the time it takes to run each participant through the study and also increases the sample size needed for statistical analysis. Lastly, our literature review did not uncover any publications demonstrating an effect of arm hair, sweat, or ambient temperature on PPG measurements (PubMed, 9/2/2020; search terms: interaction AND hair AND PPG OR pulse oximetry OR Photoplethysmograph; interaction AND sweat AND PPG OR pulse oximetry OR Photoplethysmograph; interaction AND temperature AND PPG OR pulse oximetry OR Photoplethysmograph). For practical study implementation, we recommend demonstrably quantifiable covariates with a literature-based justification for their inclusion in the study design.

The current gold standard of measuring skin tone is the Fitzpatrick Phototype (FP) Skin Type Scale, which divides the spectrum of skin tones into six ordered categories. We fully agree that there are inherent issues with both the visual assessment of skin tone and with the FP scale specifically, which was not developed initially for the full spectrum of skin tones^11^. The later addition to FP of two darker skin tone categories underscores that FP must be treated as an ordinal rather than interval variable in analysis. However, we disagree with the premise that there is no value in visual assessment methods and with the assertion that it is well established that FP has weak correlation with skin color. Specifically, two of the three references cited in the MA to support this claim do not actually assess FP^8,9^. We explored the works cited by these three references and found just one study of 43 Thai volunteers used to support the references’ claims^10,11^. On the other hand, multiple studies in diverse populations have shown that skin color evaluation with a spectrophotometer is correlated with visual skin tone assessment^12–14^.

An interesting idea proposed in the MA is to replace subjective skin tone scales with objective reflectance spectrometry. Next, we discuss the pros and cons of spectrometry versus visual skin tone assessment and make recommendations based on our own study experience. Spectrometry benefits from objective technology-based measurements, however, it requires specialized equipment and the measurements can be affected by not only skin tone, but also by tissue composition (e.g., tissue hydration status^15^). While spectrometry can reduce subjectivity associated with commonly used visual skin tone assessment, there is an increased cost of collecting and analyzing spectrometry data. We believe that requiring its use increases the barrier to entry for including skin tone as a variable in wearables accuracy studies and may thus limit the number of future studies performed in this space, which is in opposition to both our own and the MA author’s objectives. We do believe that new technology development may lower this barrier to entry. Best practices^16^ for spectrophotometry measures and potential sources of error (e.g., whether other tissue components aside from melanin content can affect spectrometry measurements) will need to be established for consistent and comparable assessment across studies^17^.

On the other hand, visual scales are lower cost, more accessible, and therefore more commonly used in research studies. However, using visual scales properly requires a trained research technician to perform all study measurements, a single printed reference color palette used for every observation, and consistent ambient lightning. Visual scales may also be subject to administrator bias. In both objective and subjective measurement methods, human error may be introduced through improper measurement methodology.

In the original study, we used both objective hand-held spectrophotometer measurements (LinkSquare, Stratio Inc) as well as two separate and independently assessed subjective visual assessments using the FP and von Luschan skin tone scales (Supplementary Table 10). For every visual assessment, the same printed FP and von Luschan scale color palette was used by the same study administrator in the same room with the same lighting. Each color swatch was placed on the wrist location where the smart watch sensor would lay, and the closest color match was chosen by the study administrator. In the original publication, we only reported the data from the two visual assessment methods (FP vs. von Luschan Spearman correlation 0.98, *p* = 2.2e−16) because we did not find differences in the spectrometry measurements across even the most divergent skin tone groups (FP1 vs. FP6; nonsignificant pairwise Wilcoxon Rank Sum Test), indicating that there was either an equipment error or that there are other factors involved in the spectrometry measurements such as tissue content that do not relate only to skin tone. This was a limitation in our original study that may be addressed through using well-evaluated equipment commonly used for objective skin tone measurements.

Recently, others have demonstrated success with objective skin tone measurements using spectrocolorimetry with the Chromasphere® (L’Oréal, Paris, France) and Datacolor microflash spectrocolorimeter (Datacolor, Montreuil, France) tools^18,19^, which differ from the spectrophotometry method we used by Linksquare Stratio Inc. This presents a potential alternative approach for objective measurements of skin tone and has been recently utilized in a study for VO_2_max accuracy in smartphones across skin tones^19^.

Our goals are aligned with the MA author ‘to fully and accurately represent the possible limitations of PPG technology for individuals with dark skin to limit any unintentional contributions to health disparities’. We hope that by providing a framework for including skin tone as a variable in device accuracy assessments and by piloting this methodology on five of the leading wearables used currently in clinical research, we have demonstrated that this is an important and accessible analysis. A key area that we believe is critical for the future of this field is to broaden the application of this study framework to all devices used in clinical research under a wider variety of circumstances of use, so that we can better capture the range of real-world scenarios where data would be collected and used for clinical or research purposes. It is also critical to continue this research as hardware and software continue to evolve, and to understand how software updates can change accuracy of wearable device measurements. Wearables companies and/or researchers using these products can and should use this methodology in appropriately powered, Institutional Review Board-approved studies to evaluate the accuracy of their devices across the full spectrum of skin tones.
